# Self‐Reported Items That Predict the Risk of Oral Health Deterioration and the Need for Dental Referral in Older People: A Systematic Review

**DOI:** 10.1111/ger.12812

**Published:** 2025-02-11

**Authors:** M. H. S. de Jong, C. D. van der Maarel‐Wierink, J. C. F. Ket, K. Jerković‐Ćosić, F. R. Rozema

**Affiliations:** ^1^ Department of Oral Medicine Academic Centre for Dentistry Amsterdam (ACTA) Amsterdam the Netherlands; ^2^ Department of Oral and Maxillofacial Surgery, Amsterdam UMC University of Amsterdam Amsterdam the Netherlands; ^3^ Medical Library Vrije Universiteit Amsterdam Amsterdam the Netherlands; ^4^ Department of Public Health and Oral Health Care Academic Centre for Dentistry Amsterdam (ACTA) Amsterdam the Netherlands; ^5^ Research Group Innovations in Preventive Care University of Applied Sciences Utrecht Utrecht the Netherlands

## Abstract

**Background:**

Detecting deterioration in frail oral community‐dwelling older people's oral health may be delayed as a consequence of decreased visits to oral health care professionals. Older people are becoming increasingly dependent on medical care and visit other healthcare professionals, highlighting the importance of interprofessional collaboration. There is a need for an easy‐to‐use, time‐ and cost‐efficient oral health assessment tool for non‐oral healthcare professionals. This systematic review aimed to identify self‐reported items that predict the risk of oral health deterioration in older people to inform such a tool.

**Method:**

The OVID/Medline, Embase, EBSCO/CINAHL, and Web of Science databases were systematically searched. An additional reference check was performed to ensure that no records were missing. The primary outcome was predictive value, defined as the probability of a specific question or self‐reported item predicting the risk of oral health deterioration or the need for dental referral. When available, the data were presented as sensitivity, specificity, positive predictive value (PPV) and negative predictive value (NPV).

**Results:**

The initial search resulted in 2471 records. Eleven articles met the inclusion criteria and were analysed. A high predictive value for oral health deterioration was observed for the self‐reported items: “Are you generally pleased with your mouth and teeth?” (specificity: 93.0%), “Would you say your mouth health is generally good?” (specificity: 95.2%), “Does your mouth feel dry?” (specificity: 82.7%), and “Do you have regular dental checkups?” (sensitivity: 90.0%–100%); and “Do you have tooth and/or mouth problems that make it hard to eat?” (specificity: 92.0).

**Conclusion:**

A screening tool for use by non‐oral health professionals, that consists of 2–4 highly predictive self‐reported items, such as dry mouth, satisfaction with oral health, recent dental visits and food consumption problems, could be used for early detection and timely referral of older people at risk of oral health deterioration.

## 1 Background

Poor oral health is associated with reduced quality of life and can negatively affect general well‐being, which is particularly salient in older people [1, 2, 3]. Since poor oral health can lead to discomfort and challenges in social life for older people, early detection of suboptimal oral health is important to ensure timely and appropriate care [4, 5]. Maintaining good oral health is crucial for preserving oral function and quality of life [6].

The number of frail older dentate people living at home is increasing, in part due to the promotion of ageing in place, and also the improved retention of natural teeth into old age. Further, as people are living longer, their morbidity, frailty and dependency on care correspondingly increases. Chronic diseases, impaired physical mobility, depressive symptoms and cognitive impairment are predictors of frailty [7]. These factors influence daily oral care, oral health and ability to visit an oral healthcare professional and, consequently, often result in oral health deterioration in frail community‐dwelling older people [8]. Detecting this deterioration is frequently delayed, thereby presenting healthcare professionals with complex care issues, including oral healthcare [9]. That requires an interprofessional approach. The growing need for oral healthcare in frail community‐dwelling older patients underlines the importance of collaboration between oral and non‐oral healthcare professionals, general practitioners, medical specialists, nurses, paramedics and other healthcare professionals.

The frequency of dental visits declines with age in older people [10, 11]. Less than 70% of adults aged 65 and older and less than 50% of adults aged 80 and older visit the dentist once a year [12, 13, 14]. Frail older people have an increased need to receive medical and nursing care. For example, more than 75% of adults aged 75 years and older visit a general practitioner at least once a year [15, 16]. This finding illustrates the growing emphasis on the role of medical staff, including general practitioners, in the oral healthcare of frail older people and further underlines the importance of interprofessional collaboration.

Most general practitioners acknowledge the importance of oral healthcare. Nevertheless, several barriers to providing oral care exist, including time and financial constraints, the absence of referral pathways and limited oral health knowledge and training [17]. Only 30% of general practitioners and nurses report having adequate knowledge and skills in oral care for frail older people [18]. It would appear that an adequate interprofessional infrastructure for managing oral health in community‐dwelling older people does not yet exist [19, 20].

Several questionnaires and oral health assessment tools exist to assist non‐oral healthcare professionals in assessing a patient's oral health status [21]. Previous reviews have investigated the accuracy and measurement properties of these tools and found that they are of good methodological quality [22, 23, 24]. Nonetheless, the adoption of these tools by general practitioners and other non‐oral healthcare professionals remains limited because of the aforementioned barriers [25, 26]. They typically require oral examinations and knowledge on how to conduct them, which demands a considerable amount of time to complete. A concise tool comprising only two or three self‐report items could provide a viable solution. A time‐ and cost‐efficient tool accessible to non‐oral health professionals and implementable in daily practice is needed for predicting the risk of oral health deterioration. To develop such a tool, identifying self‐reported items and questions that predict the risk of oral health deterioration is crucial.

In addition to using self‐reported patient items to assess oral health deterioration, integrating other social and health risk factors when assessing the risk of oral health deterioration is essential. Marchini et al. [27] conducted a literature review as part of the development of a teaching tool to assess risk factors for rapid oral health deterioration in older people. The authors found that the predictors of rapid oral health deterioration were general health, including cognitive deficits, medication use, chronic disease, limited social support and oral conditions, including reduced ability to perform oral hygiene and stopping visits to dentists.

This study aimed to address the gap in accessible assessment tools for non‐oral health professionals by identifying self‐reported items applicable in daily practice that were predictive of the risk of oral health deterioration and the need for referral in older people. Given the limited oral health knowledge among these professionals and time constraints in general practice, a concise tool that requires no oral examination is essential. The findings of this systematic review will inform the development of an easily applicable tool to enable earlier detection and timely dental referrals.

## 2 Methods

### 2.1 Design

A systematic literature review was conducted in accordance with the Preferred Reporting Items for Systematic Reviews and Meta‐Analyses (PRISMA) Statement [28].

### 2.2 Literature Search

A comprehensive literature search was performed using the following databases (from inception): OVID/Medline (up to July 8, 2022), Embase.com, EBSCO/Cumulative Index to Nursing and Allied Health Literature (CINAHL), and Clarivate Analytics/Web of Science Core Collection (up to October 7, 2022), in collaboration with a medical information specialist (JCFK). The search included controlled terms and free text terms for the synonyms of “aged” or “frail elderly” and “surveys” or “questionnaires” and “oral health” or “mouth diseases” or “jaw diseases.” The search was conducted without restrictions on the methodology, date, or language. The full search strategies can be found in Appendix S1. Duplicate articles were excluded by a medical information specialist (JCFK) using Endnote X20.0.1 (Clarivate, London, United Kingdom), following the Amsterdam Efficient Deduplication (AED) method [29] and the Bramer method [30]. In addition, references to relevant articles were reviewed.

### 2.3 Study Selection and Study Inclusion Criteria

Two researchers (M.J. and S.R.) independently reviewed the studies, first based on their titles and abstracts, followed by a full‐text review to assess their eligibility. If the researchers did not agree with the data in a selected paper, a third researcher (C.W.) was consulted to reach a consensus. Duplicate records were removed. Articles were included if they met the following criteria: (1) they were an original article; (2) they included questions and self‐reported items that predicted the risk of oral health deterioration and the need for dental referral; (3) accessibility by non‐oral healthcare professionals; (4) implementability in daily care; and (5) they included frail older people. Review articles, case series, non‐English or Dutch articles and studies that did not compare questions or self‐reported items using an oral assessment or validated screening tool were excluded.

### 2.4 Outcome

The primary outcome was the predictive value, defined as the probability of a specific question or self‐reported item predicting the risk of oral health deterioration or the need for dental referral. When available, the data were presented as sensitivity, specificity, positive predictive value (PPV), or negative predictive value (NPV).

### 2.5 Quality Assessment

The methodological quality of the included studies was assessed using the Joanna Briggs Institute Critical Appraisal Checklist for Analytical Cross‐Sectional Studies [31] and followed the same process of independent evaluation by two researchers (M.J. and S.R.) with a third researcher (C.W.) consulted in cases of disagreement. The critical appraisal tool consisted of questions about selection, comparability, and outcomes. A score was assigned, based on the various responses. Articles were scored on a scale ranging from 0 (i.e., low quality) to 8 (i.e., high quality). Articles with scores between 0 and 4 were defined as “low quality”; scores 5 and 6, as “moderate quality”; and scores 7 and 8, as “high quality.”

### 2.6 Data Extraction

The following information was extracted from the included studies: first author, year of publication, study design, patient population (including mean age) and outcomes, as previously described.

### 2.7 Statistical Considerations

If sensitivity, specificity, PPV, and NPV were not directly presented in the articles but the data necessary to determine the predictive value were available, then sensitivity, specificity, PPV, and NPV were calculated, based on the following formulas: Sensitivity = [*a*/(*a* + *c*)] × 100; Specificity = [*d*/(*b* + *d*)] × 100; PPV = [*a*/(*a* + *b*)] × 100; and NPV = [*d*/(*c* + *d*)] × 100, in which a = true positive, b = false positive, c = false negative and d = true negative. Owing to the nature and considerable heterogeneity of the included studies, the results could not be directly compared, and no additional statistical analyses were conducted.

## 3 Results

### 3.1 Study Selection

**FIGURE 1:**
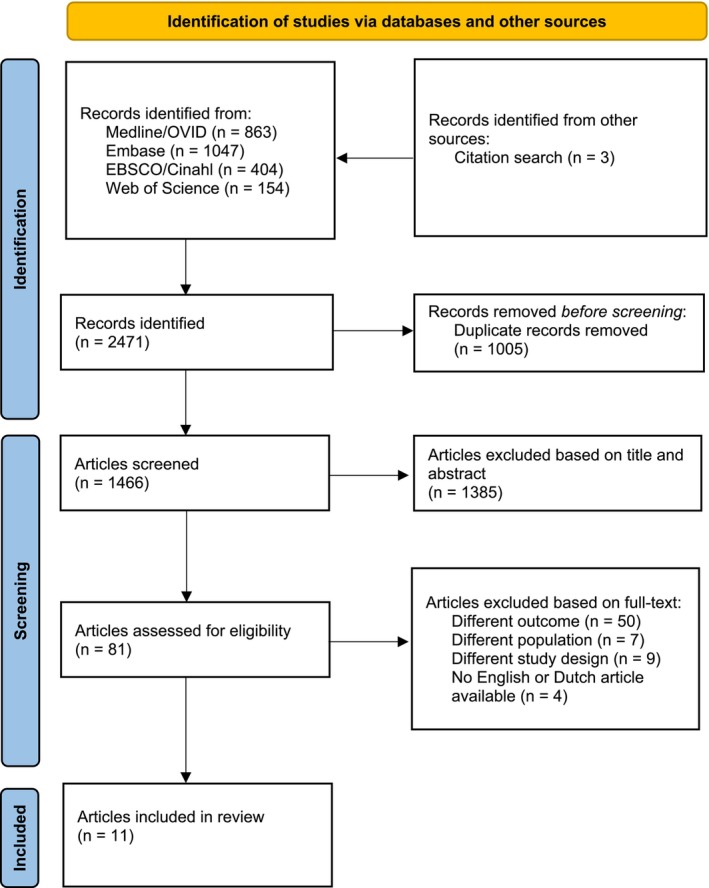
Flowchart of study inclusion. [Colour figure can be viewed at wileyonlinelibrary.com]

In the initial search, 2471 records were identified. Three eligible articles were retrieved via the citation search. After removing duplicate records, 1466 articles remained. After screening titles and abstracts, 1385 articles were excluded. The remaining 81 articles were then retrieved for full‐text evaluation. Of these, 11 studies met the inclusion criteria. Figure 1 shows a flowchart of the inclusion process.

### 3.2 Characteristics of the Included Studies

The included articles were published between 1990 and 2019. All studies had a cross‐sectional design. The number of patients included in the studies ranged from 41 patients to 1960 patients. Eight study population groups consisted of community‐dwelling older people [32, 33, 34, 35, 36, 37, 38, 39]; one study population group consisted of adults and older people seen at a dental school clinic [40]; and two study group populations consisted of community‐dwelling older people and people living in long‐term care [41, 42]. For the gold standard comparison, seven studies used clinical oral examinations [33, 35, 37, 38, 39, 40, 42], one study used self‐reported oral status [34], and three studies used one of the following oral health assessment tools: Oral Health Impact Profile‐14 (OHIP‐14), Geriatric Oral Health Assessment Index (GOHAI), or Brief Oral Health Status Examination (BOHSE) [32, 36, 41]. Eight studies reported the sensitivity, specificity, PPV, NPV, or the available data to determine this as an outcome [33, 34, 35, 36, 37, 38, 39, 40]. One study reported the percentage of normative need and care workers' assessed need as an outcome [42]. One study reported the median GOHAI and OHIP scores [41]. Another study reported the mean OHIP score [32]. Table 1 presents the overview of the included studies.

**TABLE 1:** Overview of the included studies.

Author	Year	Patient group	Self‐reported items	Comparison	Key results/predictive value
Koistinen et al. [38]	2019	Older people (> 65 years) in short‐term care (*N* = 391)	‐ Are you generally pleased with your mouth and teeth?	Clinical oral assessment	Sensitivity: 17.1%, Specificity: 93.0%
Wiener et al. [37]	2010	Community dwelling older people (> 70 year) (*N* = 252)	‐ Does your mouth feel dry?	Clinical oral assessment	Sensitivity: 40.6%, Specificity: 82.7%
Bush et al. [35]	1996	Older people (> 65 year) who visit general medicine clinic (*N* = 165)	‐ Do you have a dry mouth? ‐ Do you have difficulty eating? ‐ Did you have recent dental care (last two year)? ‐ Do you have tooth or mouth pain? ‐ Did you alter or change your food selection? ‐ Do you have lesions, sores or lumps in your mouth?	Clinical oral assessment	Sensitivity: 82.1%, Specificity: 89.6%
Hoad‐Reddick [42]	1990	Community dwelling and residential older people (*N* = 41)	‐ Do you think you need any dental treatment? ‐ Do you have any painful areas in your mouth? ‐ Do you have any problems eating? ‐ When did you last visit a dentist (last five year)?	Clinical oral assessment	Normative need for referral: 85.4% Perceived need: 36.5% Care worker assessed need (based on questions): 82.9%
Slade [39]	2007	Community dwelling older people (> 75‐year, small group of > 55 year) (*N* = 1960)	‐ Have you lost any fillings or do you need a dental visit? ‐ Have you had pain in your mouth while chewing? ‐ Have you had to interrupt meals because of problems with your teeth, mouth or dentures? ‐ Have you had difficulty relaxing because of problems with your teeth, mouth or dentures? ‐ Have you avoided laughing or smiling because of problems with your teeth, mouth or dentures?	Clinical oral assessment (medical personel)	Two affirmative responses Sensitivity: 97.6%, Specificity: 40.9% One affirmative response Sensitivity: 91.5%, Specificity: 35.2%
Drake et al. [33]	1990	Community dwelling older people (> 65 year) (*N* = 1018)	‐ As compared to others your own age, would you say that your mouth health is generally good? Or generally bad?	Clinical oral assessment; pocket depth, gingival recession, CPITN, caries, need for extraction	CPITN (maximum gingival pocket depth > 6 mm) Sensitivity: 17.6%, Specificity: 95.2%
Myers‐Wright et al. [40]	2018	Adults and older people seen at a dental school clinic seeking initial care examinations (*N* = 391)	‐ How would you rate the health of your teeth and gums (poor‐excellent)? ‐ Would you say that in general your health is (poor‐excellent)?	Clinical oral assessment; missing teeth, DMFT, probing depth > 5 mm	One question Sensitivity: 59%–71%, Specificity: 49%–56% Two questions Sensitivity: 76%–91%, Specificity: 28%–33%
Jensen et al. [32]	2008	Disabled, cognitive intact, community‐dwelling older people (> 65 year) (*N* = 641)	‐ Do you need dental treatment? ‐ Do you have a dry mouth?	OHIP‐14 [ref]	Perceived dental treatment need (no vs. yes): Mean OHIP‐14 score (SD): 1.18 (2.06) versus 3.27 (4.45), *p* < 0.001 Dry mouth (no vs. yes): Mean OHIP‐14 score (SD): 1.40 (2.48) versus 2.47 (3.86), *p* < 0.001
Locker et al. [41]	2005	Community dwelling older people and people (> 50 year) living in a geriatric setting (*N* = 766)	‐ Are you dissatisfied with your oral health status?	OHIP‐14 [ref] and GOHAI [ref]	Median GOHAI score (no vs. yes): 16–19 versus 6–10, *p* < 0.001 Median OHIP score (no vs. yes): 4–14 versus 1–7, *p* < 0.001
Chia‐Hui Chen et al. [36]	2007	Community dwelling older people (> 60 year) (*N* = 240)	‐ Do you have regular dental checkups (last year)?	BOHSE [ref] and GOHAI [ref]	GOHAI as gold standard Sensitivity: 100%, Specificity: 36.5% BOHSE as gold standard Sensitivity: 90.0%, Specificity: 35.9%
Fedele et al. [34]	1998	Older people (> 50 year) admitted to the medical ward (*N* = 300)	‐ Do you have tooth and/or mouth problems that make it hard to eat? ‐ Do you take three or more different prescribed or over‐the‐counter drugs per day? ‐ Do you have an illness or condition that changed the type or amount of food?	Self‐reported oral health status	Tooth/mouth problems: Sensitivity: 30.2%, Specificity: 92.0% Three or more drugs: Sensitivity: 56.2%, Specificity: 50.7% Illness or condition: Sensitivity: 59.3%, Specificity: 43.5%

### 3.3 Methodological Quality

The quality of the studies ranged from 5 points to 8 points, with a median score of 7 points and a mean score of 6.7 points. Four studies were of moderate quality [34, 35, 39, 42] and seven studies were of high quality [32, 33, 36, 37, 38, 40, 41]. Ten studies (90.9%) clearly defined the inclusion criteria [32, 34, 35, 36, 37, 38, 39, 40, 41, 42], identified confounding factors [32, 33, 34, 35, 36, 37, 38, 39, 40, 41], and validated reliable outcomes [32, 33, 35, 36, 37, 38, 39, 40, 41, 42]. Only three studies (27.3%) provided strategies to address confounding factors [33, 38, 41]. Eight studies (72.7%) employed the appropriate statistical analysis [32, 33, 34, 36, 37, 38, 40, 41].

### 3.4 Self‐Reported Items

An overview of the self‐reported items that predicted the risk of oral health deterioration and the need for dental referral is shown in Table 2. The following self‐reported items within the eight domains were identified as follows. Four studies included questions about ***satisfaction with oral health*** [33, 38, 40, 41]. High specificity (93.0%–95.2%) and low sensitivity (17.1%–17.6%) were reported. Poorer oral health and significantly higher median GOHAI scores (“no” vs. “yes:” 16–19 vs. 6–10, *p* < 0.001) and OHIP scores (“no” vs. “yes”: 4–14 vs. 1–7, *p* < 0.001) were reported when the participants answered negatively. Three studies included questions about ***dry mouth*** [32, 35, 37]. Moderately high specificity (82.7%) and low sensitivity (40.6%) were reported. Poorer oral health and significantly higher OHIP scores (“no” vs. “yes”: 1.40 vs. 2.47, *p* < 0.001) were reported when the participants answered positively. Three studies included questions about **patients' *perceived need*** [32, 42]. Poorer oral health and significantly higher OHIP scores (3.27 vs. 1.18, *p* < 0.001) were reported when the participants answered positively. Three studies included questions about ***recent dental visits*** [35, 36, 42]. Low specificity (35.9%–36.5%) and high sensitivity (90.0%–100%) were reported. Four studies included questions about food ***consumption problems*** [34, 35, 39, 42]. High specificity (92.0%) and low sensitivity (30.2%) were reported. Two studies included questions about ***oral pain*** [35, 42]. However, these studies only included the outcomes of a combined assessment of multiple questions. One study included a question about ***recent polypharmacy*** [34]. Low specificity (50.7%) and sensitivity (56.2%) were reported. One study included a question about a topic different from the aforementioned statements and was thus classified as “***other problems”*** [34]. This study included only the outcomes of a combined assessment of multiple questions. Appendix S3 (Supplementary Table) shows the specificity, sensitivity, PPV and NPV, based on available data from the included articles.

**TABLE 2:** Overview of the self‐reported items that predict the risk of oral health deterioration.

Topic	Question (individual)	Article	Evidence
Satisfaction with oral health	Are you dissatisfied with your oral health status?	Locker et al. [41]	Median GOHAI score (no vs. yes): 16–19 versus 6–10, *p* < 0.001 Median OHIP score (no vs. yes): 4–14 versus 1–7, *p* < 0.001
	As compared to others your own age, would you say that your mouth health is generally good? Or generally bad?	Drake et al. [33]	Sensitivity: 17.6%, Specificity: 95.2%
	Are you generally pleased with your mouth and teeth?	Koistinen et al. [40]	Sensitivity: 17.1%, Specificity: 93.0%
	How would you rate the health of your teeth and gums (poor‐excellent)?	Myers‐Wright et al. [40]	Sensitivity: 76%–91%,^a^ Specificity: 28%–33%
	Would you say that in general your health is (poor‐excellent)?	Myers‐Wright et al. [40]	Sensitivity: 76%–91%,^a^ Specificity: 28%–33%
Dry mouth	Does your mouth feel dry?	Wiener et al. [38]	Sensitivity: 40.6%, Specificity: 82.7%
	Do you have a dry mouth?	Jensen et al. [32]	Mean OHIP‐14 score (SD): 1.40 (2.48) versus 2.47 (3.86), *p* < 0.001
	Do you have a dry mouth?	Bush et al. [35]	Sensitivity: 82.1%,^a^ Specificity: 89.6%
Perceived need	Do you need dental treatment?	Jensen et al. [32]	Mean OHIP‐14 score (SD): 1.18 (2.06) versus 3.27 (4.45), *p* < 0.001
	Do you think you need any dental treatment?	Hoad‐Reddick [42]	Normative need for referral: 85.4%^a^ Perceived need: 36.5% Care worker assessed need: 82.9%
	Have you lost any fillings or do you need a dental visit?	Slade [39]	Sensitivity: 91.5%,^a^ Specificity: 35.2%
Recent dental visit	Do you have regular dental checkups (last year)?	Chia‐Hui Chen et al. [36]	GOHAI as gold standard Sensitivity: 100%, Specificity: 36.5% BOHSE as gold standard Sensitivity: 90.0%, Specificity: 35.9%
	When did you last visit a dentist (last five year)?	Hoad‐Reddick [42]	Normative need for referral: 85.4%^a^ Perceived need: 36.5% Care worker assessed need: 82.9%
	Did you have recent dental care (last two year)?	Bush et al. [35]	Sensitivity: 82.1%,^a^ Specificity: 89.6%
Food consumption problems	Have you had to interrupt meals because of problems with your teeth, mouth or dentures?	Slade [39]	Sensitivity: 91.5%,^a^ Specificity: 35.2%
	Do you have any problems eating?	Hoad‐Reddick [42]	Normative need for referral: 85.4%^a^ Perceived need: 36.5% Care worker assessed need: 82.9%
	Do you have difficulty eating?	Bush et al. [35]	Sensitivity: 82.1%,^a^ Specificity: 89.6%
	Do you have tooth and/or mouth problems that make it hard to eat?	Fedele et al. [34]	Sensitivity: 30.2%, Specificity: 92.0%
	have you had pain in your mouth while chewing?	Slade [39]	Sensitivity: 91.5%,^a^ Specificity: 35.2%
	Did you alter or change your food selection?	Bush et al. [35]	Sensitivity: 82.1%,^a^ Specificity: 89.6%
	Do you have an illness or condition that changed the type or amount of food?	Fedele et al. [34]	Sensitivity: 59.3%, Specificity: 43.5%
Oral pain	Do you have tooth or mouth pain?	Bush et al. [35]	Sensitivity: 82.1%,^a^ Specificity: 89.6%
	Do you have any painful areas in your mouth?	Hoad‐Reddick [42]	Normative need for referral: 85.4%^a^ Perceived need: 36.5% Careworker assessed need: 82.9%
	Do you have lesions, sores or lumps in your mouth?	Bush et al. [35]	Sensitivity: 82.1%,^a^ Specificity: 89.6%
Medication use	Do you take three or more different prescribed or over‐the‐counter drugs per day?	Fedele et al. [34]	Sensitivity: 56.2%, Specificity: 50.7%
Other problems	Have you had difficulty relaxing because of problems with your teeth, mouth or dentures?	Slade [39]	Sensitivity: 91.5%,^a^ Specificity: 35.2%
	Have you avoided laughing or smiling because of problems with your teeth, mouth or dentures?	Slade [39]	Sensitivity: 91.5%,^a^ Specificity: 35.2%

^a^ Combined assessment of multiple questions.

## 4 Discussion

To the best of our knowledge, this systematic review is the first to identify self‐reported patient items that can be used by non‐oral health professionals to predict the risk of oral health deterioration and need for dental referral in frail older people. Among the studies included in this review, low‐to‐moderate predictive values were found for patients' self‐reported items about perceived need and medication use. Self‐reported items had a high predictive value in the following domains: satisfaction with oral health, dry mouth, recent dental visits and food consumption problems. These findings suggested that a concise tool that focuses on these domains can accurately predict oral health deterioration in the daily practice of general practitioners and other non‐oral healthcare professionals.

This review had several strengths, including its systematic approach, focus on the predictive value of patients' self‐reported items and emphasis on items that are explicitly accessible and implementable in the daily practice of general practitioners and other non‐oral healthcare professionals.

This study also had some limitations. Not all included studies reported predictive values for sensitivity and specificity. For example, some studies provided outcomes such as the mean and median OHIP score or normative versus perceived need, thereby making direct comparisons challenging [32, 41, 42]. Further, the studies varied in methodology: some used combined assessments, whereas others relied on self‐reported health status without oral examinations. For instance, Slade et al. [39] did not clarify the training of the personnel conducting assessments, and Fedele et al. [34] did not include oral examinations. This lack of consistency complicates the interpretation of the results. In addition, the included studies varied in age classifications of the older populations. For example, Hoad‐Reddick et al. [40] did not specify age groups, whereas Myers‐Wright et al. [42] included adults and older individuals without differentiation.

Interestingly, the perceived need had a moderate‐to‐low predictive value, whereas satisfaction with oral health had a high predictive value. An assumption may be that patients' perceived need for dental care reflects their awareness about their oral health status and it predicts deterioration. However, studies have reported mixed results. Some studies have shown that the perceived need for dental care seems to align with the objective oral health status, whereas other studies have shown no such correlation [8, 43, 44]. This finding highlights the complexity of self‐assessed needs and emphasises the importance of including objective parameters such as a recent dental visit. Further, it demonstrates the need to validate and test the psychometric properties of a multi‐item tool.

The findings of this study were consistent with those of earlier studies, thereby reinforcing the potential value of self‐reported items for use by non‐oral health professionals. For instance, the interRAI Long‐Term Care Facility Instrument and the guidelines for promoting oral health in frail older people by Kossioni et al. included self‐reported items about “*dry mouth*,” “*difficulty chewing*,” “*mouth pain and problems*,” and “*recent dental visit*”, identifying these items as among the most reliable [45, 46, 47]. This overlap further supports the high predictive value of these factors in assessing the risk of oral health deterioration.

This systematic literature review identified self‐reported items with a high predictive value for oral health deterioration. These items should be combined into an implementable tool suitable for clinical settings. To ensure time efficiency while maintaining clinical relevance, the tool should include the minimal number of questions necessary to achieve high predictive accuracy. Further research is required to test this tool, determine the optimal number of questions, refine its content and assess its feasibility and acceptability.

## 5 Conclusion

A screening tool for use by non‐oral health professionals, that consists of two to four highly predictive self‐reported items, such as dry mouth, satisfaction with oral health, recent dental visits and food consumption problems, could be used for early detection and timely referral of older people at risk of oral health deterioration. Future research should focus on developing this tool, building on the groundwork laid out in this review, and validating its predictive value in clinical settings.

## Author Contributions

This work was carried out in collaboration among all authors. M.H.S.d.J. contributed to the manuscript by drafting the majority of the text and incorporating received feedback. CW also contributed significantly to the writing process, provided valuable insights regarding the study's design, and offered critical feedback. K.J.‐Ć. and F.R.R. contributed to the writing, gave crucial feedback, and provided strategic advice on the study's design. J.C.F.K. was instrumental in conducting the literature search and provided key advice for the methods section. All authors read and approved the final manuscript.

## Conflicts of Interest

The authors declare no conflicts of interest.

## Supporting information


Appendix S1:

Appendix S2:

Appendix S3:

## Data Availability

The data supporting the findings of this study are available in the Appendix of this document. All relevant datasets and materials have been included.
