# Supplementary material for: Self‐Reported Items That Predict the Risk of Oral Health Deterioration and the Need for Dental Referral in Older People: A Systematic Review
Source: Gerodontology. 2025 Feb 11;42(3):296–306. doi: 10.1111/ger.12812 (PMC12344619; doi:10.1111/ger.12812)
Supplement: Supplementary file 1 — Appendix S1: [file GER-42-296-s003.docx]

**Appendix 1.** Search

**Search strategy for Ovid/MEDLINE (8 July 2022)**

| **No.** | **Query** | **Results** |
| --- | --- | --- |
| **1** | exp Aged/ or exp "Aged, 80 and over"/ or exp Frail Elderly/ or exp Geriatrics/ or exp Geriatric Psychiatry/ or exp Geriatric Nursing/ or exp Geriatric Dentistry/ or exp "Dental Care for Aged"/ or exp "Health Services for the Aged"/ or (elder* or eldest or frail* or geriatri* or old-age* or oldest-old* or senior* or senium or very-old* or septuagenarian* or octagenarian* or octogenarian* or nonagenarian* or centarian* or centenarian* or supercentenarian* or older-people or older-subject* or older-patient* or older-age* or older-adult* or older-man or older-men or older-male* or older-woman or older-women or older-female* or older-population* or older-person* or advanced-age*).ti,ab,kf. | **3656195** |
| **2** | "Surveys and Questionnaires"/ or exp Patient Reported Outcome Measures/ or exp Self Report/ or (survey* or patient-report* or self-report*).ti,ab,kf. | **1299971** |
| **3** | exp Oral Health/ or exp Oral Hygiene/ or exp Mouth Diseases/ or exp Jaw Diseases/ or exp Tooth Diseases/ or (tooth or teeth or dental* or dentin* or edentulous or malocclusi* or mal-occlusi* or caries or carious* or cariogeni* or odontalgi* or cervical-car* or anodonti* or periapical* or peri-apical* or periodontiti* or white-spot* or stomatit* or oromucositi* or ((mouth or oral or gingiva* or jaw or salivar* or palatal or palatum or stomatognathic* or pulp or maxilla* or mandibul* or temporomandibular*) adj3 (health* or hygien* or diseas* or disorder* or syndrom* or hemorrhag* or haemorrhag* or fistul* or mucositi* or implantiti* or ulcer* or fibrosi* or cyst* or pulpiti* or fluori* or hypercementosi* or infect* or candidias* or thrush* or monilias* or leukoedema* or leuko-edema* or leukooedema* or leuko-oedema* or leakag* or lichen* or submucos* or granulom*)) or ((root or roots) adj3 (fractur* or avulsi* or loss or lost or resorpt* or injur* or decay* or infect* or sensitiv*))).ti,ab,kf. | **856065** |
| **4** | exp "Referral and Consultation"/ or (patient-need* or refer* or consult* or oral-assessment* or dental-assessment*).ti,ab,kf. | **1133382** |
| **5** | 1 and 2 and 3 and 4 | **863** |

**Search strategy for Embase.com (7 October 2022)**

| **No.** | **Query** | **Results** |
| --- | --- | --- |
| **1** | 'aged'/exp OR 'geriatrics'/exp OR 'elderly care'/exp OR elder*:de,ab,ti,kw OR eldest:de,ab,ti,kw OR frail*:de,ab,ti,kw OR geriatri*:de,ab,ti,kw OR ((old NEXT/1 age*):de,ab,ti,kw) OR ((oldest NEXT/1 old*):de,ab,ti,kw) OR senior*:de,ab,ti,kw OR senium:de,ab,ti,kw OR ((very NEXT/1 old*):de,ab,ti,kw) OR septuagenarian*:de,ab,ti,kw OR octagenarian*:de,ab,ti,kw OR octogenarian*:de,ab,ti,kw OR nonagenarian*:de,ab,ti,kw OR centarian*:de,ab,ti,kw OR centenarian*:de,ab,ti,kw OR supercentenarian*:de,ab,ti,kw OR 'older people':de,ab,ti,kw OR ((older NEXT/1 subject*):de,ab,ti,kw) OR ((older NEXT/1 patient*):de,ab,ti,kw) OR ((older NEXT/1 age*):de,ab,ti,kw) OR ((older NEXT/1 adult*):de,ab,ti,kw) OR 'older man':de,ab,ti,kw OR 'older men':de,ab,ti,kw OR 'older male*':de,ab,ti,kw OR 'older woman':de,ab,ti,kw OR 'older women':de,ab,ti,kw OR 'older female*':de,ab,ti,kw OR ((older NEXT/1 population*):de,ab,ti,kw) OR ((older NEXT/1 person*):de,ab,ti,kw) OR 'advanced-age*':de,ab,ti,kw | **3971527** |
| **2** | 'questionnaire'/exp OR 'patient-reported outcome'/de OR' self report'/exp OR 'survey'/exp OR survey*:ti,ab,kw OR 'patient-report*':ti,ab,kw OR 'self-report*':ti,ab,kw | **1915394** |
| **3** | 'mouth hygiene'/exp OR 'mouth disease'/exp OR 'jaw disease'/exp OR tooth:ti,ab,kw OR teeth:ti,ab,kw OR dental*:ti,ab,kw OR dentin*:ti,ab,kw OR edentulous:ti,ab,kw OR malocclusi*:ti,ab,kw OR 'mal-occlusi*':ti,ab,kw OR caries:ti,ab,kw OR carious*:ti,ab,kw OR cariogeni*:ti,ab,kw OR odontalgi*:ti,ab,kw OR 'cervical-car*':ti,ab,kw OR anodonti*:ti,ab,kw OR periapical*:ti,ab,kw OR 'peri-apical*':ti,ab,kw OR periodontiti*:ti,ab,kw OR 'white-spot*':ti,ab,kw OR stomatit*:ti,ab,kw OR oromucositi*:ti,ab,kw OR (((mouth OR oral OR gingiva* OR jaw OR salivar* OR palatal OR palatum OR stomatognathic* OR pulp OR maxilla* OR mandibul* OR temporomandibular*) NEAR/3 (health* OR hygien* OR diseas* OR disorder* OR syndrom* OR hemorrhag* OR haemorrhag* OR fistul* OR mucositi* OR implantiti* OR ulcer* OR fibrosi* OR cyst* OR pulpiti* OR fluori* OR hypercementosi* OR infect* OR candidias* OR thrush* OR monilias* OR leukoedema* OR 'leuko-edema*' OR leukooedema* OR 'leuko-oedema*' OR leakag* OR lichen* OR submucos* OR granulom*)):ti,ab,kw) OR (((root OR roots) NEAR/3 (fractur* OR avulsi* OR loss OR lost OR resorpt* OR injur* OR decay* OR infect* OR sensitiv*)):ti,ab,kw) | **1118707** |
| **4** | 'patient referral'/exp OR 'patient-need*':ti,ab,kw OR refer*:ti,ab,kw OR consult*:ti,ab,kw OR 'oral-assessment*':ti,ab,kw OR 'dental-assessment*':ti,ab,kw | **1683942** |
| **5** | #1 AND #2 AND #3 AND #4 | **1197** |
| **6** | #5 AND ('Conference Abstract'/it OR 'Conference Paper'/it) | **150** |
| **7** | #5 NOT #6 | **1047** |

**Search strategy for EBSCO/CINAHL (7 October 2022)**

| **No.** | **Query** | **Results** |
| --- | --- | --- |
| **1** | MH "Aged+" OR MH "Aged, 80 and Over" OR MH "Frail Elderly" OR MH "Geriatrics" OR MH "Geriatric Psychiatry" OR MH "Gerontologic Nursing+" OR MH "Gerontologic Care" OR MH "Health Services for the Aged" OR TI(elder* OR eldest OR frail* OR geriatri* OR "old age*" OR "oldest old*" OR senior* OR senium OR "very old*" OR septuagenarian* OR octagenarian* OR octogenarian* OR nonagenarian* OR centarian* OR centenarian* OR supercentenarian* OR "older people" OR "older subject*" OR "older patient*" OR "older age*" OR "older adult*" OR "older man" OR "older men" OR "older male" OR "older woman" OR "older women" OR "older female" OR "older population*" OR "older person*") OR AB(elder* OR eldest OR frail* OR geriatri* OR "old age*" OR "oldest old*" OR senior* OR senium OR "very old*" OR septuagenarian* OR octagenarian* OR octogenarian* OR nonagenarian* OR centarian* OR centenarian* OR supercentenarian* OR "older people" OR "older subject*" OR "older patient*" OR "older age*" OR "older adult*" OR "older man" OR "older men" OR "older male" OR "older woman" OR "older women" OR "older female" OR "older population*" OR "older person*") OR KW(elder* OR eldest OR frail* OR geriatri* OR "old age*" OR "oldest old*" OR senior* OR senium OR "very old*" OR septuagenarian* OR octagenarian* OR octogenarian* OR nonagenarian* OR centarian* OR centenarian* OR supercentenarian* OR "older people" OR "older subject*" OR "older patient*" OR "older age*" OR "older adult*" OR "older man" OR "older men" OR "older male" OR "older woman" OR "older women" OR "older female" OR "older population*" OR "older person*") | **1,042,557** |
| **2** | MH "Questionnaires+" OR MH "Self Report+" OR MH "Surveys+" OR TI(survey* OR "patient-report*" OR "self-report*") OR AB(survey* OR "patient-report*" OR "self-report*") OR KW(survey* OR "patient-report*" OR "self-report*") | **901,204** |
| **3** | MH "Oral Hygiene+" OR MH "Mouth Care" OR MH "Mouth Diseases+" OR MH "Jaw Diseases+" OR MH "Jaw Neoplasms+" OR MH "Jaw Cysts+" OR MH "Odontogenic Cysts+" OR MH "Mandibular Diseases+" OR MH "Craniomandibular Disorders+" OR MH "Maxillary Diseases+" OR MH "Mouth Abnormalities+" OR MH "Mouth, Edentulous+" OR MH "Mouth Neoplasms+" OR MH "Leukoplakia, Oral+" OR MH "Salivary Gland Neoplasms+" OR MH "Mucositis+" OR MH "Oral Fistula+" OR MH "Tongue Diseases+" OR MH "Stomatitis+" OR MH "Salivary Gland Diseases+" OR MH "Periodontal Diseases+" OR MH "Gingival Diseases+" OR MH "Periodontal Atrophy+" OR MH "Periodontitis+" OR MH "Parotid Diseases+" OR MH "Xerostomia+" OR MH "Tooth Diseases+" OR MH "Bruxism+" OR MH "Dental Deposits+" OR MH "Malocclusion+" OR MH "Tooth Abnormalities+" OR MH "Tooth Demineralization+" OR MH "Tooth Injuries+" OR MH "Tooth Resorption+" OR TI(tooth OR teeth OR dental* OR dentin* OR edentulous OR malocclusi* OR "mal-occlusi*" OR caries OR carious* OR cariogeni* OR odontalgi* OR "cervical-car*" OR anodonti* OR periapical* OR "peri-apical*" OR periodontiti* OR "white-spot*" OR stomatit* OR oromucositi* OR ((mouth OR oral OR gingiva* OR jaw OR salivar* OR palatal OR palatum OR stomatognathic* OR pulp OR maxilla* OR mandibul* OR temporomandibular*) N3 (health* OR hygien* OR diseas* OR disorder* OR syndrom* OR hemorrhag* OR haemorrhag* OR fistul* OR mucositi* OR implantiti* OR ulcer* OR fibrosi* OR cyst* OR pulpiti* OR fluori* OR hypercementosi* OR infect* OR candidias* OR thrush* OR monilias* OR leukoedema* OR "leuko-edema*" OR leukooedema* OR "leuko-oedema*" OR leakag* OR lichen* OR submucos* OR granulom*)) OR ((root OR roots) N3 (fractur* OR avulsi* OR loss OR lost OR resorpt* OR injur* OR decay* OR infect* OR sensitiv*))) OR AB(tooth OR teeth OR dental* OR dentin* OR edentulous OR malocclusi* OR "mal-occlusi*" OR caries OR carious* OR cariogeni* OR odontalgi* OR "cervical-car*" OR anodonti* OR periapical* OR "peri-apical*" OR periodontiti* OR "white-spot*" OR stomatit* OR oromucositi* OR ((mouth OR oral OR gingiva* OR jaw OR salivar* OR palatal OR palatum OR stomatognathic* OR pulp OR maxilla* OR mandibul* OR temporomandibular*) N3 (health* OR hygien* OR diseas* OR disorder* OR syndrom* OR hemorrhag* OR haemorrhag* OR fistul* OR mucositi* OR implantiti* OR ulcer* OR fibrosi* OR cyst* OR pulpiti* OR fluori* OR hypercementosi* OR infect* OR candidias* OR thrush* OR monilias* OR leukoedema* OR "leuko-edema*" OR leukooedema* OR "leuko-oedema*" OR leakag* OR lichen* OR submucos* OR granulom*)) OR ((root OR roots) N3 (fractur* OR avulsi* OR loss OR lost OR resorpt* OR injur* OR decay* OR infect* OR sensitiv*))) OR KW(tooth OR teeth OR dental* OR dentin* OR edentulous OR malocclusi* OR "mal-occlusi*" OR caries OR carious* OR cariogeni* OR odontalgi* OR "cervical-car*" OR anodonti* OR periapical* OR "peri-apical*" OR periodontiti* OR "white-spot*" OR stomatit* OR oromucositi* OR ((mouth OR oral OR gingiva* OR jaw OR salivar* OR palatal OR palatum OR stomatognathic* OR pulp OR maxilla* OR mandibul* OR temporomandibular*) N3 (health* OR hygien* OR diseas* OR disorder* OR syndrom* OR hemorrhag* OR haemorrhag* OR fistul* OR mucositi* OR implantiti* OR ulcer* OR fibrosi* OR cyst* OR pulpiti* OR fluori* OR hypercementosi* OR infect* OR candidias* OR thrush* OR monilias* OR leukoedema* OR "leuko-edema*" OR leukooedema* OR "leuko-oedema*" OR leakag* OR lichen* OR submucos* OR granulom*)) OR ((root OR roots) N3 (fractur* OR avulsi* OR loss OR lost OR resorpt* OR injur* OR decay* OR infect* OR sensitiv*))) | **186,981** |
| **4** | MH "Referral and Consultation" OR TI("patient-need*" OR refer* OR consult* OR "oral-assessment*" OR "dental-assessment*") OR AB("patient-need*" OR refer* OR consult* OR "oral-assessment*" OR "dental-assessment*") OR KW("patient-need*" OR refer* OR consult* OR "oral-assessment*" OR "dental-assessment*") | **324,240** |
| **5** | S1 AND S2 AND S3 AND S4 | **404** |

**Search strategy for Clarivate Analytics Web of Science Core Collection (7 October 2022)**

| **No.** | **Query** | **Results** |
| --- | --- | --- |
| **1** | **TS=("elder*" OR "eldest" OR "frail*" OR "geriatri*" OR "old age*" OR "oldest old*" OR "senior*" OR "senium" OR "very old*" OR "septuagenarian*" OR "octagenarian*" OR "octogenarian*" OR "nonagenarian*" OR "centarian*" OR "centenarian*" OR "supercentenarian*" OR "older people" OR "older subject*" OR "older patient*" OR "older age*" OR "older adult*" OR "older man" OR "older men" OR "older male" OR "older woman" OR "older women" OR "older female" OR "older population*" OR "older person*" OR "advanced age*")** | **827,991** |
| **2** | **TS=("survey*" OR "patient-report*" OR "self-report*")** | **1,673,495** |
| **3** | **TS=("tooth" OR "teeth" OR "dental*" OR "dentin*" OR "edentulous" OR "malocclusi*" OR "mal-occlusi*" OR "caries" OR "carious*" OR "cariogeni*" OR "odontalgi*" OR "cervical-car*" OR "anodonti*" OR "periapical*" OR "peri-apical*" OR "periodontiti*" OR "white-spot*" OR "stomatit*" OR "oromucositi*" OR (("mouth" OR "oral" OR "gingiva*" OR "jaw" OR "salivar*" OR "palatal" OR "palatum" OR "stomatognathic*" OR "pulp" OR "maxilla*" OR "mandibul*" OR "temporomandibular*") NEAR/3 ("health*" OR "hygien*" OR "diseas*" OR "disorder*" OR "syndrom*" OR "hemorrhag*" OR "haemorrhag*" OR "fistul*" OR "mucositi*" OR "implantiti*" OR "ulcer*" OR "fibrosi*" OR "cyst*" OR "pulpiti*" OR "fluori*" OR "hypercementosi*" OR "infect*" OR "candidias*" OR "thrush*" OR "monilias*" OR "leukoedema*" OR "leuko-edema*" OR "leukooedema*" OR "leuko-oedema*" OR "leakag*" OR "lichen*" OR "submucos*" OR "granulom*")) OR (("root" OR "roots") NEAR/3 ("fractur*" OR "avulsi*" OR "loss" OR "lost" OR "resorpt*" OR "injur*" OR "decay*" OR "infect*" OR "sensitiv*" )))** | **539,538** |
| **4** | **TS=("patient-need*" OR "refer*" OR "consult*" OR "oral-assessment*" OR "dental-assessment*")** | **1,753,548** |
| **5** | **#4 AND #3 AND #2 AND #1** | **154** |
