# Supplementary material for: Self‐Reported Items That Predict the Risk of Oral Health Deterioration and the Need for Dental Referral in Older People: A Systematic Review
Source: Gerodontology. 2025 Feb 11;42(3):296–306. doi: 10.1111/ger.12812 (PMC12344619; doi:10.1111/ger.12812)
Supplement: Supplementary file 2 — Appendix S2: [file GER-42-296-s002.docx]

|  |  | | | ***Joanna Briggs Institute Critical Appraisal Checklist for Analytical Cross-Sectional Studies*** | | | | | | | | | | | | | | | | | | | | | | | | | |  | | |  | | |  |  |
| --- | --- | --- | --- | --- | --- | --- | --- | --- | --- | --- | --- | --- | --- | --- | --- | --- | --- | --- | --- | --- | --- | --- | --- | --- | --- | --- | --- | --- | --- | --- | --- | --- | --- | --- | --- | --- | --- |
|  |  | | | |  | | | | |  | | | | | | | | |  | |  | |  | |  | |  | | | |  | | |  | | |  |
|  |  | | | | *Were the criteria for inclusion in the sample clearly defined?* | | *Were the study subjects and the setting described in detail?* | | | | | *Was the exposure measured in a valid and reliable way?* | | | | *Were objective, standard criteria used for measurement of the condition?* | | | *Were confounding factors identified?* | | *Were strategies to deal with confounding factors stated?* | | *Were the outcomes measured in a valid and reliable way?* | | *Was appropriate statistical analysis used?* | | *Overall Score* | |  |  |  |  |  |  |  |  |  |
|  | *Koistinen et al. [30]* | | | | yes | | yes | | | | | yes | | | | | yes | | yes | | yes | | yes | | yes | | 8 | |  |  |  |  |  |  |  |  |  |
|  | *Myers-Wright et al. [29]* | | | | yes | | yes | | | | | yes | | | | | yes | | yes | | unclear | | yes | | yes | | 7 | |  |  |  |  |  |  |  |  |  |
|  | *Wiener et al. [28]* | | | | yes | | yes | | | | | yes | | | | | yes | | yes | | unclear | | yes | | yes | | 7 | |  |  |  |  |  |  |  |  |  |
|  | *Jensen et al. [22]* | | | | yes | | yes | | | | | yes | | | | | yes | | yes | | unclear | | yes | | yes | | 7 | |  |  |  |  |  |  |  |  |  |
|  | *Chia-Hui Chen et al. [26]* | | | | yes | | yes | | | | | yes | | | | | yes | | yes | | unclear | | yes | | yes | | 7 | |  |  |  |  |  |  |  |  |  |
|  | *Slade [31]* | | | | yes | | yes | | | | | yes | | | | | yes | | yes | | unclear | | yes | | unclear | | 6 | |  |  |  |  |  |  |  |  |  |
|  | *Locker et al. [27]* | | | | yes | | yes | | | | | yes | | | | | yes | | yes | | yes | | yes | | yes | | 8 | |  |  |  |  |  |  |  |  |  |
|  | *Fedele et al. [24]* | | | | yes | | yes | | | | | yes | | | | | yes | | yes | | unclear | | unclear | | yes | | 6 | |  |  |  |  |  |  |  |  |  |
|  | *Bush et al. [25]* | | | | yes | | yes | | | | | yes | | | | | yes | | yes | | unclear | | yes | | unclear | | 6 | |  |  |  |  |  |  |  |  |  |
|  | *Hoad-Reddick [32]* | | | | yes | | yes | | | | | yes | | | | | yes | | unclear | | unclear | | yes | | unclear | | 5 | |  |  |  |  |  |  |  |  |  |
|  | *Drake et al. [23]* | | | | unclear | | yes | | | | | yes | | | | | yes | | yes | | yes | | yes | | yes | | 7 | |  |  |  |  |  |  |  |  |  |
|  |  | | | |  | | | | |  | | | | | | | | |  | |  | |  | |  | |  | | | |  | | |  | | |  |
|  | *** Points were given when answered "yes". For the answers "no" and "unclear" no points were given.** | | | | | | | | | | | | | | | | | | |  | |  | |  | |  | |  | | | |  | | |  | | |
|  |  | |  | | | | | |  | | | |  |  |  |  |  |  |  |  |  |  |  |  |  |  |  |  |  |  |  |  |  |  |  |  |  |
|  |  |  | | | |  | |  | | | | | |  | | | |  |  |  |  |  |  |  |  |  |  |  |  |  |  |  |  |  |  |  |  |
|  |  | |  | | | | | |  | | | |  |  |  |  |  |  |  |  |  |  |  |  |  |  |  |  |  |  |  |  |  |  |  |  |  |
|  |  |  |  |  |  |  |  |  |  |  |  |  |  |  |  |  |  |  |  |  |  |  |  |  |  |  |  |  |  |  |  |  |  |  |  |  |  |
|  |  |  | | |  | | | | | |  | | | |  |  |  |  |  |  |  |  |  |  |  |  |  |  |  |  |  |  |  |  |  |  |  |
|  |  |  | | |  | | | | | |  | | | |  |  |  |  |  |  |  |  |  |  |  |  |  |  |  |  |  |  |  |  |  |  |  |
|  |  |  | | |  | | | | | |  | | | |  |  |  |  |  |  |  |  |  |  |  |  |  |  |  |  |  |  |  |  |  |  |  |
|  |  |  | | | |  | |  | | | | | |  | | | |  |  |  |  |  |  |  |  |  |  |  |  |  |  |  |  |  |  |  |  |

**Appendix 2.** Quality assessment
