# Supplementary material for: Self‐Reported Items That Predict the Risk of Oral Health Deterioration and the Need for Dental Referral in Older People: A Systematic Review
Source: Gerodontology. 2025 Feb 11;42(3):296–306. doi: 10.1111/ger.12812 (PMC12344619; doi:10.1111/ger.12812)
Supplement: Supplementary file 3 — Appendix S3: [file GER-42-296-s001.docx]

| **Appendix 3.** Sensitivity and specificity calculations* |  | Koistinen et al. (2019) | |  | |  | |  | |  | |  | |  | |  | |  | |  |
| --- | --- | --- | --- | --- | --- | --- | --- | --- | --- | --- | --- | --- | --- | --- | --- | --- | --- | --- | --- | --- |
|  |  | Need for oral referral based on gold standard | |  | |  | |  | |  | |  | |  | |  | |  | |  |
|  |  | Yes | No | Total: | |  | |  | |  | |  | |  | |  | |  | |  |
| Need for referral based on question | Yes | 49 | 7 | 56 | |  | |  | |  | |  | |  | |  | |  | |  |
|  | No | 238 | 80 | 318 | |  | |  | |  | |  | |  | |  | |  | |  |
|  | Total: | 287 | 86 | 374 | |  | |  | |  | |  | |  | |  | |  | |  |
|  |  |  |  |  | |  | |  | |  | |  | |  | |  | |  | |  |
|  |  | Sensitivity: | 17,1% |  | |  | |  | |  | |  | |  | |  | |  | |  |
|  |  | Specificity: | 93,0% |  | |  | |  | |  | |  | |  | |  | |  | |  |
|  |  | PPV: | 87,5% |  | |  | |  | |  | |  | |  | |  | |  | |  |
|  |  | NPV: | 25,2% |  | |  | |  | |  | |  | |  | |  | |  | |  |
|  |  |  |  |  | |  | |  | |  | |  | |  | |  | |  | |  |
|  |  |  |  |  | |  | |  | |  | |  | |  | |  | |  | |  |
|  |  | Wiener et al. (2010) | |  | |  | |  | |  | |  | |  | |  | |  | |  |
|  |  | Need for oral referral based on gold standard | |  | |  | |  | |  | |  | |  | |  | |  | |  |
|  |  | Yes | No | Total: | |  | |  | |  | |  | |  | |  | |  | |  |
| Need for referral based on question | Yes | 13 | 35 | 48 | |  | |  | |  | |  | |  | |  | |  | |  |
|  | No | 19 | 167 | 186 | |  | |  | |  | |  | |  | |  | |  | |  |
|  | Total: | 32 | 202 | 234 | |  | |  | |  | |  | |  | |  | |  | |  |
|  |  |  |  |  | |  | |  | |  | |  | |  | |  | |  | |  |
|  |  | Sensitivity: | 40,6% |  | |  | |  | |  | |  | |  | |  | |  | |  |
|  |  | Specificity: | 82,7% |  | |  | |  | |  | |  | |  | |  | |  | |  |
|  |  | PPV: | 27,1% |  | |  | |  | |  | |  | |  | |  | |  | |  |
|  |  | NPV: | 89,8% |  | |  | |  | |  | |  | |  | |  | |  | |  |
|  |  |  |  |  | |  | |  | |  | |  | |  | |  | |  | |  |
|  |  |  |  |  | |  | |  | |  | |  | |  | |  | |  | |  |
|  |  | Bush et al. (1996) | |  | |  | |  | |  | |  | |  | |  | |  | |  |
|  |  | Need for oral referral based on gold standard | |  | |  | |  | |  | |  | |  | |  | |  | |  |
|  |  | Yes | No | Total: | |  | |  | |  | |  | |  | |  | |  | |  |
| Need for referral based on question | Yes | 96 | 5 | 101 | |  | |  | |  | |  | |  | |  | |  | |  |
|  | No | 21 | 43 | 64 | |  | |  | |  | |  | |  | |  | |  | |  |
|  | Total: | 117 | 48 | 165 | |  | |  | |  | |  | |  | |  | |  | |  |
|  |  |  |  |  | |  | |  | |  | |  | |  | |  | |  | |  |
|  |  | Sensitivity: | 82,1% |  | |  | |  | |  | |  | |  | |  | |  | |  |
|  |  | Specificity: | 89,6% |  | |  | |  | |  | |  | |  | |  | |  | |  |
|  |  | PPV: | 95,0% |  | |  | |  | |  | |  | |  | |  | |  | |  |
|  |  | NPV: | 67,2% |  | |  | |  | |  | |  | |  | |  | |  | |  |
|  |  |  |  |  | |  | |  | |  | |  | |  | |  | |  | |  |
|  |  |  |  |  | |  | |  | |  | |  | |  | |  | |  | |  |
|  |  | Chia-Hui Chen et al. (2007) (GOHAI) | |  | |  | |  | |  | |  | | Chia-Hui Chen et al. (2007) (BOHSE) | | | |  | |  |
|  |  | Need for oral referral based on gold standard | |  | |  | |  | |  | |  | | Need for oral referral based on gold standard | | | |  | |  |
|  |  | Yes | No | Total: | |  | |  | |  | |  | | Yes | | No | | Total: | |  |
| Need for referral based on question | Yes | 18 | 141 | 159 | |  | |  | | Need for referral based on question | | Yes | | 18 | | 141 | | 159 | |  |
|  | No | 0 | 81 | 81 | |  | |  | |  |  | No | | 2 | | 79 | | 81 | |  |
|  | Total: | 18 | 222 | 240 | |  | |  | |  | | Total: | | 20 | | 220 | | 240 | |  |
|  |  |  |  |  | |  | |  | |  | |  | |  | |  | |  | |  |
|  |  | Sensitivity: | 100,0% |  | |  | |  | |  | |  | | Sensitivity: | | 90,0% | |  | |  |
|  |  | Specificity: | 36,5% |  | |  | |  | |  | |  | | Specificity: | | 35,9% | |  | |  |
|  |  | PPV: | 11,3% |  | |  | |  | |  | |  | | PPV: | | 11,3% | |  | |  |
|  |  | NPV: | 100,0% |  | |  | |  | |  | |  | | NPV: | | 97,5% | |  | |  |
|  |  |  |  |  | |  | |  | |  | |  | |  | |  | |  | |  |
|  |  |  |  |  | |  | |  | |  | |  | |  | |  | |  | |  |
|  |  | Slade (2007) (2 affirmative response) | |  | |  | |  | |  | |  | | Slade (2007) (1 affirmative response) | | | |  | |  |
|  |  | Need for oral referral based on gold standard | |  | |  | |  | |  | |  | | Need for oral referral based on gold standard | | | |  | |  |
|  |  | Yes | No | Total: | |  | |  | |  | |  | | Yes | | No | | Total: | |  |
| Need for referral based on question | Yes | 327 | 25 | 352 | |  | |  | | Need for referral based on question | | Yes | | 281 | | 89 | | 370 | |  |
|  | No | 472 | 1027 | 1499 | |  | |  | |  |  | No | | 518 | | 963 | | 1481 | |  |
|  | Total: | 799 | 1052 | 1851 | |  | |  | |  | | Total: | | 799 | | 1052 | | 1851 | |  |
|  |  |  |  |  | |  | |  | |  | |  | |  | |  | |  | |  |
|  |  | Sensitivity: | 40,9% |  | |  | |  | |  | |  | | Sensitivity: | | 35,2% | |  | |  |
|  |  | Specificity: | 97,6% |  | |  | |  | |  | |  | | Specificity: | | 91,5% | |  | |  |
|  |  | PPV: | 92,9% |  | |  | |  | |  | |  | | PPV: | | 75,9% | |  | |  |
|  |  | NPV: | 68,5% |  | |  | |  | |  | |  | | NPV: | | 65,0% | |  | |  |
|  |  |  |  |  | |  | |  | |  | |  | |  | |  | |  | |  |
|  |  |  |  |  | |  | |  | |  | |  | |  | |  | |  | |  |
|  |  | Drake et al. (1990) | |  | |  | |  | |  | |  | |  | |  | |  | |  |
|  |  | Need for oral referral based on gold standard | |  | |  | |  | |  | |  | |  | |  | |  | |  |
|  |  | Yes | No | Total: | |  | |  | |  | |  | |  | |  | |  | |  |
| Need for referral based on question | Yes | 16 | 14 | 30 | |  | |  | |  | |  | |  | |  | |  | |  |
|  | No | 75 | 279 | 354 | |  | |  | |  | |  | |  | |  | |  | |  |
|  | Total: | 91 | 293 | 384 | |  | |  | |  | |  | |  | |  | |  | |  |
|  |  |  |  |  | |  | |  | |  | |  | |  | |  | |  | |  |
|  |  | Sensitivity: | 17,6% |  | |  | |  | |  | |  | |  | |  | |  | |  |
|  |  | Specificity: | 95,2% |  | |  | |  | |  | |  | |  | |  | |  | |  |
|  |  | PPV: | 53,3% |  | |  | |  | |  | |  | |  | |  | |  | |  |
|  |  | NPV: | 78,8% |  | |  | |  | |  | |  | |  | |  | |  | |  |
|  |  |  |  |  | |  | |  | |  | |  | |  | |  | |  | |  |
|  |  |  |  |  | |  | |  | |  | |  | |  | |  | |  | |  |
|  |  | Fedele et al. (1998) (touth/mouth problems) | |  | |  | |  | |  | |  | | Fedele et al. (1998) (condition/illness) | | | |  | |  |
|  |  | Need for oral referral based on gold standard | |  | |  | |  | |  | |  | | Need for oral referral based on gold standard | | | |  | |  |
|  |  | Yes | No | Total: | |  | |  | |  | |  | | Yes | | No | | Total: | |  |
| Need for referral based on question | Yes | 49 | 11 | 60 | |  | |  | | Need for referral based on question | | Yes | | 91 | | 68 | | 159 | |  |
|  | No | 113 | 127 | 240 | |  | |  | |  |  | No | | 71 | | 70 | | 141 | |  |
|  | Total: | 162 | 138 | 300 | |  | |  | |  | | Total: | | 162 | | 138 | | 300 | |  |
|  |  |  |  |  | |  | |  | |  | |  | |  | |  | |  | |  |
|  |  | Sensitivity: | 30,2% |  | |  | |  | |  | |  | | Sensitivity: | | 56,2% | |  | |  |
|  |  | Specificity: | 92,0% |  | |  | |  | |  | |  | | Specificity: | | 50,7% | |  | |  |
|  |  | PPV: | 81,7% |  | |  | |  | |  | |  | | PPV: | | 57,2% | |  | |  |
|  |  | NPV: | 52,9% |  | |  | |  | |  | |  | | NPV: | | 49,6% | |  | |  |
|  |  |  |  |  | |  | |  | |  | |  | |  | |  | |  | |  |
|  |  |  |  |  | |  | |  | |  | |  | |  | |  | |  | |  |
|  |  | Fedele et al. (1998) (>3 prescribed drugs) | |  | |  | |  | |  | |  | |  | |  | |  | |  |
|  |  | Need for oral referral based on gold standard | |  | |  | |  | |  | |  | |  | |  | |  | |  |
|  |  | Yes | No | Total: | |  | |  | |  | |  | |  | |  | |  | |  |
| Need for referral based on question | Yes | 96 | 78 | 174 | |  | |  | |  | |  | |  | |  | |  | |  |
|  | No | 66 | 60 | 126 | |  | |  | |  | |  | |  | |  | |  | |  |
|  | Total: | 162 | 138 | 300 | |  | |  | |  | |  | |  | |  | |  | |  |
|  |  |  |  |  | |  | |  | |  | |  | |  | |  | |  | |  |
|  |  | Sensitivity: | 59,3% |  | |  | |  | |  | |  | |  | |  | |  | |  |
|  |  | Specificity: | 43,5% |  | |  | |  | |  | |  | |  | |  | |  | |  |
|  |  | PPV: | 55,2% |  | |  | |  | |  | |  | |  | |  | |  | |  |
|  |  | NPV: | 47,6% |  | |  | |  | |  | |  | |  | |  | |  | |  |
|  |  |  |  |  | |  | |  | |  | |  | |  | |  | |  | |  |
|  |  |  |  |  | |  | |  | |  | |  | |  | |  | |  | |  |
|  |  |  |  |  | |  | |  | |  | |  | |  | |  | |  | |  |
|  |  |  |  |  | |  | |  | |  | |  | |  | |  | |  | |  |
| * For Myers-Wright et al. the reported sensitivity and specificity reported in the article is used | | | | |  | |  | |  | |  | |  | |  | |  | |  | |
|  |  |  |  |  | |  | |  | |  | |  | |  | |  | |  | |  |
